# Redox Regulation of Microvascular Permeability: IL-1β Potentiation of Bradykinin-Induced Permeability Is Prevented by Simvastatin

**DOI:** 10.3390/antiox9121269

**Published:** 2020-12-14

**Authors:** Felipe Freitas, Eduardo Tibiriçá, Mita Singh, Paul A. Fraser, Giovanni E. Mann

**Affiliations:** 1 Centre of Research Excellence, King’s College London British Heart Foundation, School of Cardiovascular Medicine & Sciences, Faculty of Life Sciences & Medicine, King’s College London, 150 Stamford Street, London SE1 9NH, UK.; f.freitas@ucl.ac.uk (F.F.); mita.singh@kcl.ac.uk (M.S.); 2 National Institute of Cardiology, Ministry of Health, Rio de Janeiro 22240-006, Brazil; etibi@uol.com.br

**Keywords:** microvascular permeability, bradykinin, interleukin 1β, NADPH oxidase, reactive oxygen species, simvastatin

## Abstract

Antioxidant effects of statins have been implicated in the reduction in microvascular permeability and edema formation in experimental and clinical studies. Bradykinin (Bk)-induced increases in microvascular permeability are potentiated by IL-1β; however, no studies have examined the protection afforded by statins against microvascular hyperpermeability. We investigated the effects of simvastatin pretreatment on albumin–fluorescein isothiocyanate conjugate (FITC-albumin) permeability in post-capillary venules in rat cremaster muscle. Inhibition of nitric oxide synthase with L-NAME (10µM) increased basal permeability to FITC-albumin, which was abrogated by superoxide dismutase and catalase. Histamine-induced (1 µM) permeability was blocked by L-NAME but unaffected by scavenging reactive oxygen species with superoxide dismutase (SOD) and catalase. In contrast, bradykinin-induced (1–100 nM) permeability increases were unaffected by L-NAME but abrogated by SOD and catalase. Acute superfusion of the cremaster muscle with IL-1β (30 pM, 10 min) resulted in a leftward shift of the bradykinin concentration–response curve. Potentiation by IL-1β of bradykinin-induced microvascular permeability was prevented by the nicotinamide adenine dinucleotide phosphate oxidase (NADPH oxidase) inhibitor apocynin (1 µM). Pretreatment of rats with simvastatin (5 mg·kg^−1^, i.p.) 24 h before permeability measurements prevented the potentiation of bradykinin permeability responses by IL-1β, which was not reversed by inhibition of heme oxygenase-1 with tin protoporphyrin IX (SnPP). This study highlights a novel mechanism by which simvastatin prevents the potentiation of bradykinin-induced permeability by IL-1β, possibly by targeting the assembly of NADPH oxidase subunits. Our findings highlight the therapeutic potential of statins in the prevention and treatment of patients predisposed to inflammatory diseases.

## 1. Introduction

Microvascular endothelial barrier disruption occurs in a large number of disease states, such as stroke, sepsis, diabetes, hereditary and acquired angioedema, commonly induced by a variety of endogenous inflammatory mediators such as bradykinin [1,2,3,4,5,6]. Novel therapeutic approaches to prevent or reduce microvascular permeability are paramount to avoid tissue edema and to maintain sufficient blood supply to target organs. In this context, statins have been described to reduce vascular permeability and edema formation in different animal and clinical studies [7,8,9,10], yet the underlying mechanisms have not been investigated in an intact muscle microvasculature. 

Bradykinin has several pathophysiological functions and activates the B2 receptor, which is constitutively expressed on the vasculature and increases vascular permeability in post-capillary venules [11]. Moreover, bradykinin is an important mediator in stroke, sepsis, diabetes, hereditary and acquired angioedema [1,2,3,4,5,6]. Bradykinin may also play a key role in the vascular leakage and pulmonary edema in patients with COVID-19 [12,13,14]. Angiotensin converting enzyme 2 (ACE2) has been implicated as the cellular receptor of SARS-CoV-2 virus [15,16], and reduced ACE2 activity may indirectly activate the kallikrein–bradykinin pathway to increase vascular permeability [17].

In vitro and in vivo studies have shown that the increase in vascular permeability induced by bradykinin depends on the generation of reactive oxygen species [18,19]. We previously reported that bradykinin-induced microvascular permeability in the brain pial microvasculature in vivo is directly associated with the release of reactive oxygen species following bradykinin receptor activation [20]. The pro-inflammatory cytokine IL-1β has been shown potentiate the actions of bradykinin and to increase microvascular permeability and edema formation after experimental cerebral ischemia reperfusion injury [19,21,22]. Under ischemic conditions, IL-1β is rapidly released from brain tissue, leading to NADPH oxidase assembly and activation, which then rapidly potentiates the permeability response to bradykinin [19]. Notably, potentiation of bradykinin-induced increases in cerebral microvascular permeability are blocked by the IL-1 receptor antagonist, IL-1ra [19]. Moreover, acute release of IL-1β has been described as a key inflammatory event in patients with COVID-19 [23,24,25] that could also potentiate bradykinin-induced vascular permeability. 

Clinical and experimental studies indicate several beneficial effects of statins independent of their cholesterol-lowering action [26,27,28]. Statins may have the potential to reduce oxidative stress by modulating Nrf2-regulated antioxidant genes [29,30], such as heme oxygenase 1 (HO-1) known to afford protection in rodent models of ischemia in vivo [31,32] and in vascular cells in vitro [29,33]. Further evidence suggests that simvastatin may upregulate HO-1 independently of Nrf2 [34].

To date there are no studies focused on the protective actions of statins against IL-1β mediated potentiation of bradykinin-induced microvascular permeability. In this study, we investigate for the first time the effects of pretreatment of rats with simvastatin on bradykinin- and IL-1β-induced microvascular permeability using intravital microscopy in an intact cremaster muscle preparation that to date has not been reported. Our findings suggest that simvastatin prevents microvascular hyperpermeability induced by IL-1β and bradykinin via inhibition of NADPH oxidase and inhibition of reactive oxygen species generation. 

## 2. Materials and Methods 

### 2.1. Animals and Isolation of the Cremaster Skeletal Muscle Preparation

This study conforms with the Guide for the Care and Use of Laboratory Animals published by the US National Institutes of Health (NIH Publication No. 85–23, revised 1996) and is in accordance with UK Home Office regulations (Animals Scientific Procedures) Act, 1986. Approved by UK Home Office Animal Project License (PPL Number: 70/8934).

Male Wistar rats (Charles River, UK), 4–6 weeks old and weighing 80–100 g), were killed by exposure to a rising concentration of CO_2_ followed by cervical dislocation. A longitudinal midline incision (1–2 cm) was made along the abdomen to expose the underlying organs. All the small branches of the aorta except the common iliac arteries leading branches that did not supply the chosen cremaster were tied off and the vena cava was then punctured to create an outlet for the blood that was flushed out of the circulation. The aorta was cannulated orthogradely with a polythene tubing (outside diameter 0.61 mm). The left common iliac and the right femoral and internal iliac arteries were ligated to ensure that perfusion was directed to the right external iliac artery supplying the cremaster artery and the cremaster muscle microvasculature. The tissue was perfused with a modified St. Thomas’ cardioplegic solution (mM: 10 MgCl_2_, 110 NaCl, 8 KCl, 1 CaCl_2_, 10 HEPES) [35] containing heparin (30 U/mL) and isoproterenol 10 µM buffered to pH 7.0 ± 0.05 for 10 min.

### 2.2. Superfusion of Cremaster Muscle Preparation

A longitudinal incision was made along the length of the ventral aspect of the scrotum and the overlying fascia and connective tissue were carefully removed. The cremaster was pulled out with the testicle using a pair of blunt forceps, and the distal end of the muscle was secured on a SylGard block using histology pins (Watkins and Doncaster, Kent, England). The intact cremaster preparation was then transferred to a modified stage of an intravital microscope (ACM, Zeiss, Oberkochen, Germany) and continuously superfused (2 mL·min^−1^) with an albumin-free Krebs solution (pH 7.4) gassed with 5% CO_2_ in air and maintained at 37 °C. The superfusate contained the Na^+^ channel blocker lidocaine (20 mg·L^−1^) to block neural activity and to minimize cremaster muscle contractions. 

### 2.3. Measurement of Post-Capillary Venule Permeability to FITC-Albumin

The stabilizing solution perfusing the cremaster vasculature was replaced with Krebs solution (mM: 118 NaCl; 4.7 KCl; 2.52 CaCl_2_; 1.18 MgSO_4_.7H_2_O; 1.18 KH_2_PO_4_; 25 NaHCO_3_, 5 glucose and buffered to pH 7.4 ± 0.05) containing bovine albumin (10 mg·mL^−1^) delivered by a gravity controlled reservoir at 0.5 mL·min^−1^. After 30 min, Krebs perfusion of the vasculature was stopped and a bolus of Krebs solution containing FITC-albumin (5 mg·mL^−1^) was injected into the perfusion line. Post-capillary venules were identified by noting the direction of flow, as the microvasculature was filled with the fluorescent dye, using a 10× water immersion objective (numerical aperture 0.5). Images were captured using an FITC filter cube (Chroma Technology Bellows Falls, VT, USA) via image-intensified CCD camera (Photonic Sciences, Robertsbridge, E. Sussex, UK) for subsequent analysis (ImageHopper; Samsara Research, Dorking, Surrey, UK). 

**Figure 1:**
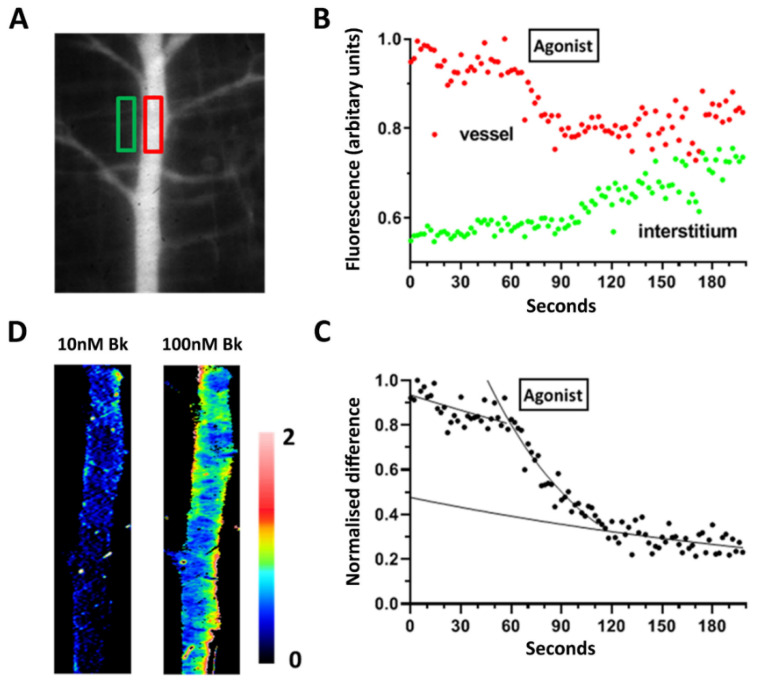
Basal and agonist stimulated permeability measurements in single venules in a rat cremaster muscle preparation. (**A**) Representative fluorescence image of the cremaster microvasculature following arterial FITC-albumin infusion. A sequence of images was captured at 1 s intervals after all axial flow had ceased, during which time (70–110 s, panel **B**) either histamine or bradykinin was applied topically. (**B**) Image stack was analyzed by placing regions of interest (ROIs) over the 33 µm diameter venule (red) and the neighboring interstitium (green). (**C**) Difference between these values for the two ROI yields the albumin concentration gradient across the microvessel. The rate constant (k) for the fitted monoexponential and the diameter gives the permeability value P = kD/4, assuming a circular diameter. (**D**) A few venules, such as the one illustrated in panel A, were on the surface of the cremaster, not overlaid with skeletal muscle fibers, which allowed a color-coded permeability map to be generated: the scale values are expressed as cm·s^−1^ × 10^−6^. The left-hand image was generated following application of 10 nM bradykinin and the right-hand image after 100 nM bradykinin.

Perfusion pressure was lowered to atmospheric, and pressure differences in the vasculature were allowed to dissipate over the course of 3 min. In previous studies, we have demonstrated a linear correlation between the light collected with the dye concentration and as well as with the square of the diameter of the microvessel up to a 60 µm limit [36]. Permeability measurements were obtained from an image sequence acquired at 1 s intervals over 100 s. The dye concentration difference across a vessel was calculated from the difference between the regions of interest positioned on an image stack (see Figure 1A,B). Permeability was determined from the rate of decrease in that difference, obtained by fitting an exponential to the data (Figure 1C) such that P = kD/4, where k is the rate constant and r is the vessel diameter. The lack of axial flow under the experimental conditions was confirmed by viewing fluorescent microspheres (1 µm diameter) within the vasculature (data not shown). It was possible to generate a permeability map on a few occasions when the venule was on the exposed surface of the cremaster preparation, so that there was no overlying tissue and that any escaped dye dissipated rapidly. Under these circumstances, the rate constants could be calculated on a pixel by pixel basis during the exponential fall of dye (see Figure 1D) by taking linear regression of the log (V-I), where V and I are the pixel values within the vessel and the interstitium, respectively.

### 2.4. Role of Nitric Oxide and Reactive Oxygen Species in Microvascular Permeability

To determine the role of nitric oxide and reactive oxygen species on basal permeability, the cremaster preparation was superfused for 5 min with a nitric oxide synthase (NOS) inhibitor, N-ω-nitro-L-arginine methyl ester (L-NAME; 10 µM) and/or the free radical scavengers superoxide dismutase (SOD, 100 U·mL^−1^) and catalase (CAT, 100 U·mL^−1^). Further experiments examined the effects of the vasoactive mediators histamine (1 µM) and bradykinin (100 nM) on permeability in the absence or presence of L-NAME or SOD and CAT.

### 2.5. Bradykinin- and IL-1β-Induced Increases in Microvascular Permeability 

Increasing concentrations of bradykinin (10^−9^, 10^−8^ and 10^−7^ M) were applied abluminally to the cremaster muscle to elicit dose dependent increases in FITC-albumin permeability. After a dose–response curve to bradykinin, the cremaster muscle was rapidly superfused and IL-1β (30 pM) applied abluminally for 10 min. The preparation was then superfused to remove IL-1β, and a new dose–response curve to bradykinin (10^−9^, 10^−8^ and 10^−7^ M) performed. To reduce variability between drug applications, the same region of the cremaster microvasculature was observed throughout an entire experiment, paired permeability measurements obtained in single post-capillary venules.

### 2.6. Inhibition of NADPH Oxidase Assembly

To determine the involvement of NADPH oxidase in the microvascular hyperpermeability induced by IL-1β and bradykinin, the cremaster preparation was superfused for 10 min with IL-1β (30 pM) in the presence of apocynin (Apo, 1 µM), a specific inhibitor of NADPH oxidase in control rats as well as in simvastatin pretreated rats. The preparation was then rapidly superfused to remove IL-1β and apocynin and bradykinin (100 nM) applied abluminally.

### 2.7. Pretreatment of Animals with Simvastatin

Simvastatin (5 mg·kg^−1^) was administered to rats intraperitoneally 24 h before isolation of the cremaster muscle preparation. 

### 2.8. Inhibition of Heme Oxygenase-1 with Tin Protophoryrin IX (SnPP)

The HO-1 inhibitor, tin protoporphyrin IX (SnPP) (5 µM), was applied abluminally for 10 min. The preparation was then superfused to remove the (SnPP), and bradykinin (100 nM) was applied. The cremaster muscle was then rapidly superfused (washed) and IL-1β (30 pM) was co-applied with (SnPP) (5 µM) abluminally for 10 min. The preparation was then superfused to remove IL-1β and (SnPP) and bradykinin (100 nM) applied abluminally.

### 2.9. Reagents

All chemicals were purchased from Sigma-Aldrich (Dorset, UK). 

### 2.10. Statistical Analysis

Experimental data represent paired permeability measurements in single venules from different animals or are expressed as mean ± SEM of measurements in single venules from *n* = 4–10 animals. Data were analyzed using a paired or unpaired Student’s *t*-test and ANOVA in GraphPad Prism 6.0 (La Jolla, CA, USA), with *p* <0.05 considered statistically significant. 

## 3. Results

### 3.1. Role of NO and Reactive Oxygen Species in Modulating Basal Microvascular Permeability

**Figure 2:**
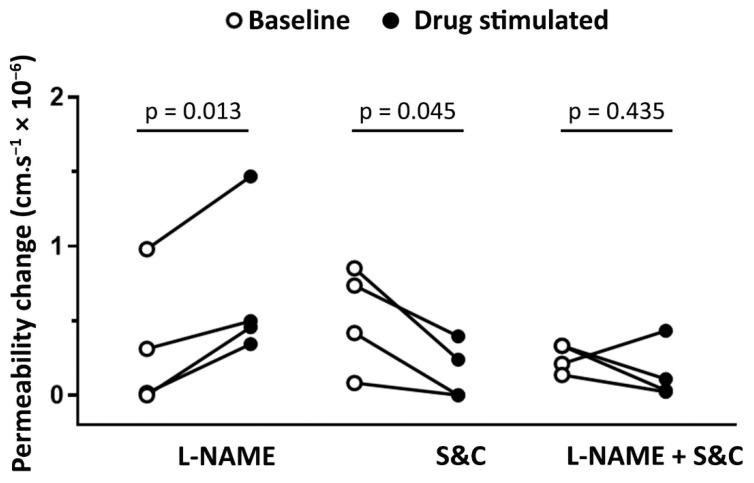
Constitutive nitric oxide reduces basal post-capillary venule permeability. Inhibiting constitutive eNOS with L-NAME (10 µM) resulted in a significant permeability increase, while scavenging reactive oxygen species with a combination superoxide dismutase and catalase (100 U·mL^−1^ each) reduced basal permeability. When superoxide dismutase and catalase were co-applied with L-NAME, there was no permeability change. Data from paired measurements in 4 venules from 4 different animals. Data were analyzed using a paired Student’s *t*-test.

Application of the nitric oxide synthase inhibitor L-NAME (10 µM) increased permeability (0.69 ± 0.26 cm·s^−1^ × 10^−6^, *p* < 0.05) above basal levels (0.33 ± 0.23 cm·s^−1^ × 10^−6^, Figure 2). Notably, co-application of superoxide dismutase (SOD, 100 U·mL^−1^) and catalase (CAT, 100 U·mL^−1^) with L-NAME abrogated the permeability increase (0.15 ± 0.09 cm·s^−1^ × 10^−6^ vs. 0.25 ±0.05 cm·s^−1^ × 10^−6^, Figure 2) evoked by L-NAME. 

### 3.2. Histamine- and Bradykinin-Induced Microvascular Permeability Is Mediated by Different Signaling Pathways

**Figure 3:**
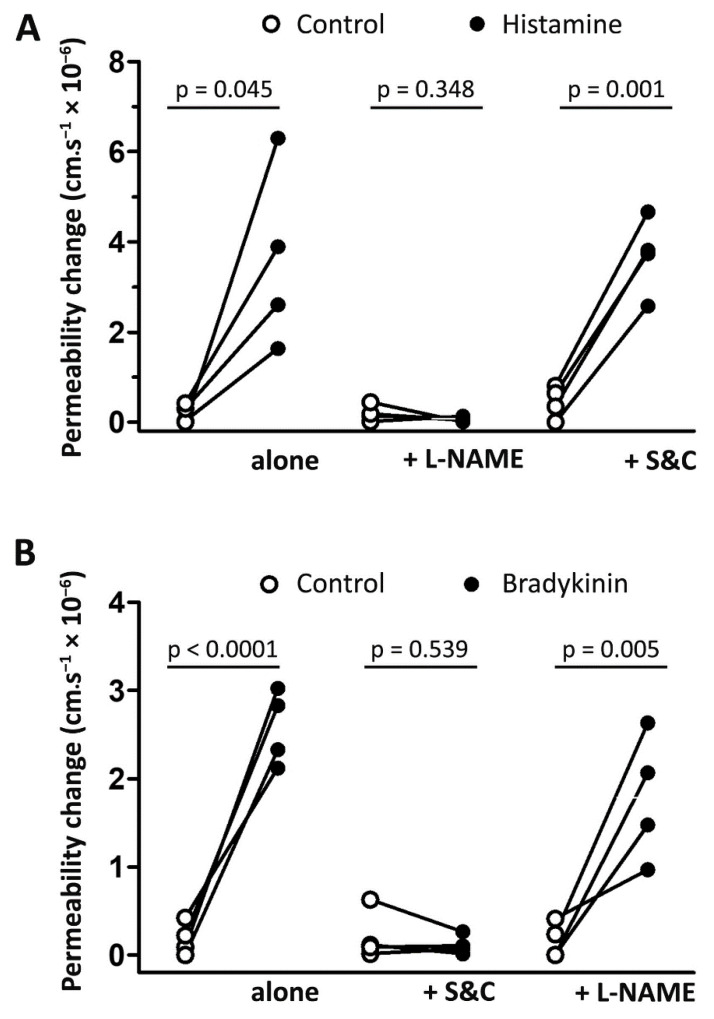
Involvement of reactive oxygen species and nitric oxide in histamine- and bradykinin-induced permeability. Changes in venular permeability following application of histamine (**A**) or bradykinin (**B**) at 1 µM in the absence or presence of superoxide dismutase (SOD, 100 U·mL^−1^) and catalase (CAT, 100 U·mL^−1^) or L-NAME (10 µM). Data from paired measurements in 4 venules from 4 different animals. Data were analyzed using a paired Student’s *t*-test.

Histamine-induced (1 µM) permeability increases (3.4 ± 1.0 cm·s^−1^ × 10^−6^) were blocked by L-NAME (−0.1 ± 0.1 cm·s^−1^ × 10^−6^) but unaffected by the free radical scavengers superoxide dismutase and catalase (Figure 3A). In contrast, as shown in Figure 3B, the permeability increase induced by bradykinin (100 nM, 2.2 ± 0.2 cm·s^−1^ × 10^−6^) was unaffected by L-NAME (1.6 ± 0.4 cm·s^−1^ × 10^−6^) but blocked by co-application of superoxide dismutase and catalase (100 U·mL^−1^; −0.1 ± 0.1 cm·s^−1^ × 10^−6^). 

### 3.3. Bradykinin-Induced Microvascular Permeability Is Potentiated by IL-1β

**Figure 4:**
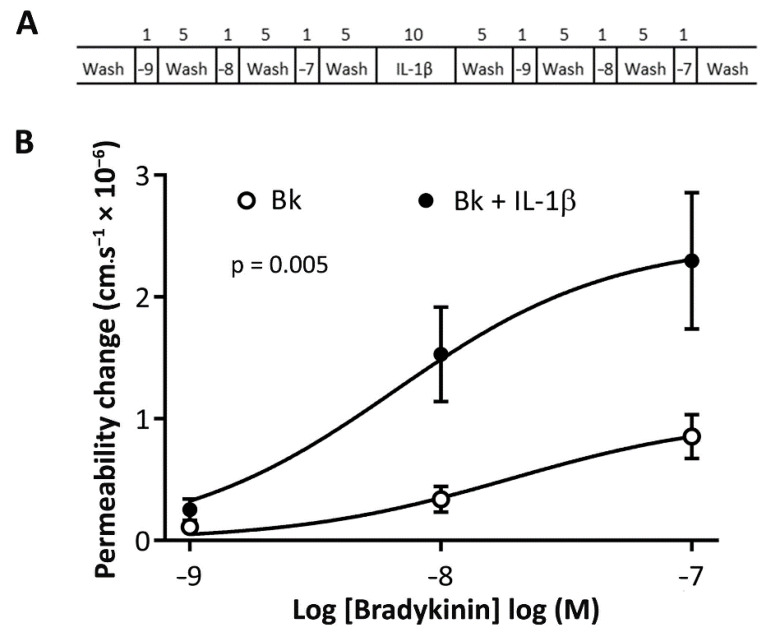
Acute treatment with IL-1β potentiates bradykinin-induced microvascular permeability. (**A**) Experimental protocol for dose–response curves to bradykinin (Bk) in the presence of kininase inhibitors. Bradykinin dose–response curves were obtained in the absence of IL-1β and after acute application of IL-1β (30 pM) for 10 min, followed by wash-off and reapplication of bradykinin applications to the same post-capillary venule. The numbers in panel A indicate the time in minutes for each phase of the protocol. (**B**) Bradykinin permeability response curve following IL-1β preapplication was significantly greater than all other responses. Data denote mean ± SEM of measurements in 8 vessels from 8 different animals, repeated measures, analysis of co-variance.

Bradykinin applied abluminally to the cremaster microcirculation induced a dose-dependent increase in permeability to FITC-albumin (Figure 4A,B). Acute treatment with IL-1β (30 pM) for 10 min, followed by wash-off of IL-1β, resulted in a significant potentiation of bradykinin-induced permeability responses (Figure 4B).

### 3.4. A Role for NADPH Oxidase and Reactive Oxygen Species in the Potentiation of Bradykinin-Induced Microvascular Permeability by IL-1β

**Figure 5:**
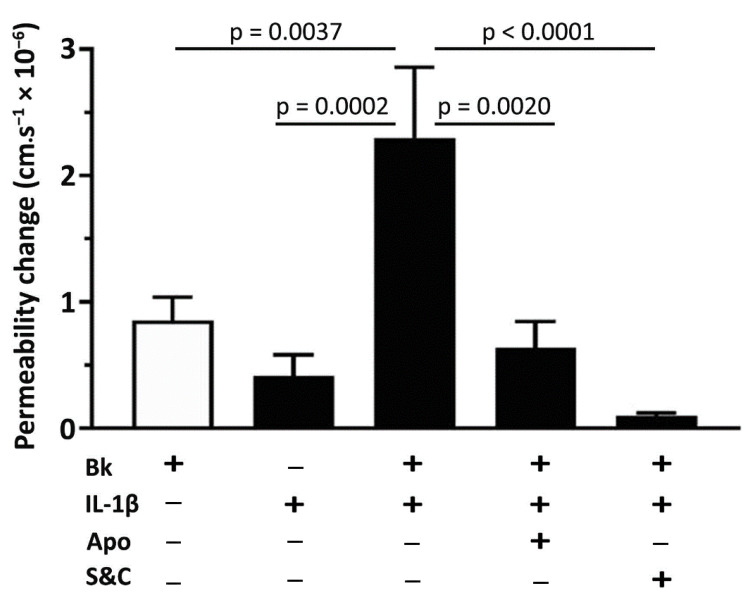
IL-1β potentiates bradykinin-induced microvascular permeability and involves NADPH oxidase and reactive oxygen species. The potentiated response to bradykinin (100 nM) application, following application of IL-1β (30 pM, 10 min), was prevented by co-application of apocynin (1 µM) with IL-1β (30 pM). Scavenging reactive oxygen species with superoxide dismutase (100 U/mL) and catalase (100 U/mL) completely blocked the permeability response to bradykinin. Data denote mean ± SEM, n = 10 venules from 10 animals. One-way ANOVA with Tukey’s multiple comparison test.

Figure 5 summarizes changes in permeability obtained in single post-capillary venules in response to bradykinin (100 nM), IL-1β (30 pM), or bradykinin (100 nM) following 10 min treatment with IL-1β (30 pM). Apocynin, co-applied with IL-1β, effectively prevented the potentiation of bradykinin-induced permeability (Figure 5). Free radical scavenging by a mixture of by superoxide dismutase and catalase completely blocked the permeability response to bradykinin following IL-1β. 

### 3.5. Pretreatment of Animals with Simvastatin

**Figure 6:**
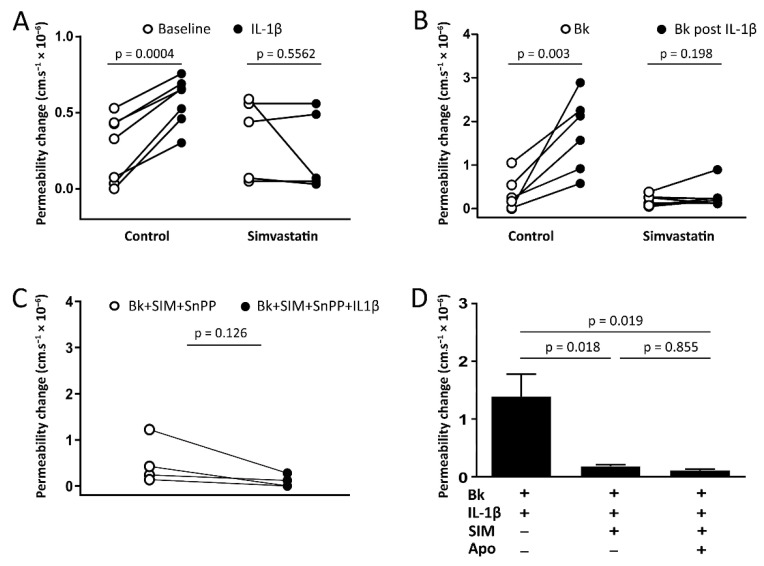
Pretreatment with simvastatin abolishes potentiation of bradykinin-induced microvascular permeability by IL-1β. (**A**) IL-1β (30 pM) application itself for 10 min resulted in a small permeability increase in non-treated rats (control), which was abrogated by the pretreatment with simvastatin (5 mg·mL^−1^) 24 h before. (**B**) Potentiation of bradykinin-induced (100 nM) permeability by IL-1β (30 pM) was compared in cremaster muscle post-capillary venules from control and simvastatin pretreated (5 mg/kg; i.p.) animals. (**C**) Inhibition of HO-1 with SnPP (5 µM) did not restore IL-1β potentiation of bradykinin-induced permeability in simvastatin pretreated (5 mg/kg; i.p.) animals. Data were analyzed using a paired Student’s *t*-test. (**D**) Apocynin had no effect on simvastatin-treated animals, suggesting that the pretreatment with simvastatin was sufficient to prevent the assembly of NADPH oxidase induced by IL-1β. One-way ANOVA with Tukey’s multiple comparison test. Data denote mean ± SEM of paired measurements in 3–6 venules from 4–6 different animals in each group.

Figure 6A demonstrates that in non-treated animals, application of IL-1β (30 pM, 10 min) in the absence of bradykinin resulted in a small permeability increase, which was prevented by the pretreatment of animals with simvastatin (5 mg·mL^−1^) 24 h before. In subsequent experiments, animals were pretreated with simvastatin (5 mg·mL^−1^) 24 h before an acute application of IL-1β (30 pM), followed by wash-off of IL-1β and application of bradykinin (100 nM). Pretreatment with simvastatin did not alter hyperpermeability induced by bradykinin alone (*p* = 0.411; Figure 6B). As shown in Figure 6B, pretreatment with simvastatin abolished the potentiation of bradykinin-induced microvascular permeability by IL-1β, with no significant effect on the permeability response to bradykinin alone. To examine whether the simvastatin induced loss of IL-1β potentiation of the bradykinin permeability response was due to an upregulation of HO-1, the cremaster preparation was treated with tin protophoryrin IX (SnPP), a known inhibitor of HO-1 [37,38]. Notably, inhibition of HO-1 with SnPP could not restore IL-1β mediated potentiation of bradykinin-induced permeability (Figure 6C). Apocynin (1 µM), a specific inhibitor of NADPH oxidase, also had no effect in simvastatin pretreated animals, suggesting that pretreatment with simvastatin was sufficient to prevent the assembly of NADPH oxidase induced by IL-1β (Figure 6D).

## 4. Discussion

The present study in an intact skeletal muscle microvasculature provides the first evidence that simvastatin prevents small permeability increases induced by IL-1β alone, as well as IL-1β mediated potentiation of bradykinin-induced microvascular permeability, highlighting the importance of pleiotropic effects of statins. Importantly, inhibition of Nox2 assembly by apocynin [37] or scavenging of reactive oxygen species with superoxide dismutase and catalase abolished the microvascular hyperpermeability induced by IL-β and bradykinin, strongly implicating Nox2 mediated free radical generation in increased microvascular permeability.

Our study confirms our previous findings in cerebral microvessels in vivo that acute bradykinin application results in a reactive oxygen species mediated increase in microvascular permeability. We report here that basal skeletal muscle microvascular permeability is reduced by scavenging reactive oxygen species, and that an increased permeability observed following inhibition of nitric oxide generation is abrogated by superoxide dismutase and catalase (Figure 2). This finding indicates that constitutive NO generation effectively scavenges basal formation of reactive oxygen species. There are numerous indications in the literature that NOS inhibition exacerbates inflammatory conditions, and this may provide an explanation for this. 

Bradykinin-induced microvascular permeability has been associated with increased NO production and vasodilation [39,40], and a key role for reactive oxygen species generated following bradykinin receptor activation has been reported in cultured endothelial cells in vitro [18,41] and in rat cerebral microvessels in vivo [19]. Further studies in vivo, using scavengers of reactive oxygen species, confirmed these findings and showed that superoxide generation contributed to the vasodilation [42] and increased permeability following bradykinin application [19,20]. Similar to these findings, we have shown that bradykinin-induced permeability in rat cremaster muscle post-capillary venules was inhibited by superfusion with superoxide dismutase and catalase (Figure 3B). In addition, the fact that L-NAME did not inhibit bradykinin-induced permeability in cremaster muscle venules argues against a role for NO and supports findings in rat mesentery [43] and brain [20].

Histamine has been shown to increase cGMP production in endothelial cells via endothelial derived NO production, with increased vascular permeability and vasodilation mediated via activation of soluble guanylyl cyclase [44,45]. In this context, treatment of the cremaster muscle preparation with L-NAME allowed us to establish that histamine-induced permeability increases were NO-dependent but unaffected by scavenging of reactive oxygen species. 

Although intracellular signaling pathways underlying reactive oxygen species mediated permeability increases were not studied, it is likely that bradykinin may induce permeability changes via the generation of free radicals during arachidonic acid metabolism leading to Ca^2+^ entry through areas of lipid peroxidation, as we previously reported for brain pial microvessels in vivo [20]. The attenuation of bradykinin-induced permeability responses in the presence of superoxide dismutase and catalase suggests that bradykinin-induced permeability increases are linked to free radical generation in rat cremaster muscle. This finding is consistent with previous reports from our laboratory that permeability responses to bradykinin in the brain microvasculature in vivo involve the generation of reactive oxygen species [19,20].

Statins have been described to improve endothelial function, reduce vascular permeability and edema formation in different experimental and clinical studies [9,46,47,48,49,50]. A clinical study with hypercholesterolemic patients assessed transcapillary albumin escape rate as an index of macromolecular permeability, and notably simvastatin treatment over 1 month normalized increases in transvascular albumin leakage independently of lipid levels in these patients [51]. Using an Evans blue dye exclusion test, simvastatin treatment for 1 month reduced vascular leakage in the aorta of hyperlipidemic rabbits [52]. Moreover, simvastatin treatment for 5 weeks improves endothelial barrier permeability changes in the brain, retina and myocardium of streptozotocin-induced diabetes rats [53]. 

Notably, administration of simvastatin 24 h before and along with intratracheal injection of lipopolysaccharide (LPS) attenuates vascular leak and inflammation in a murine inflammatory model of acute lung injury [7]. Simvastatin reduced approximately 50% of albumin levels in the bronchoalveolar lavage, and leakage of albumin conjugated with Evans blue dye into the pulmonary parenchyma in a murine inflammatory lung injury model [7]. Additionally, acute oral administration of simvastatin reduces brain edema formation and blood–brain barrier permeability after traumatic brain injury in rats [9]. In a model of experimental intracerebral hemorrhage in rats, simvastatin treatment increases cerebral blood flow in the injured region of the brain and reduces blood-brain barrier (BBB) permeability and cerebral edema [10]. Simvastatin also acutely protects the neurovascular unit, reducing blood–brain barrier permeability, when administered subcutaneously 30 min after transient cerebral ischemia induced by middle cerebral artery occlusion [8]. It is important, however, to highlight that most of these previous studies evaluated permeability changes using indirect methods, such as the Evans blue dye test. Our findings establish that simvastatin has the potential to protect the endothelial barrier and reduce vascular permeability; however, further studies are necessary to elucidate the mechanisms involved in these processes and measuring permeability coefficients.

It has been reported that lovastatin induces expression of bradykinin type 2 receptors in cultured human coronary artery endothelial cells [54]. However, in order to confirm these in vitro findings, additional in vivo studies with statin treatment in humans and in animal models are required. Simvastatin was chosen in the present study based on its potency and pharmacokinetic properties. The potency rank order for HMG-CoA reductase inhibition among the second-generation statins is simvastatin > pravastatin > lovastatin ≅ mevastatin [55]. Furthermore, lipophilic statins, such as simvastatin, are considered more likely to enter endothelial cells by passive diffusion in contrast to hydrophilic statins, such as pravastatin and rosuvastatin, which are primarily targeted to the liver [56]. Hydrophilic statins have been described to exert similar effects on the vasculature to lipophilic statins suggesting that specific mechanisms may exist for the uptake of the former; however, this may take longer than the lipophilic statins [57].

Bradykinin has been shown to play a key role in blood–brain barrier disruption and edema formation in different pathophysiological processes, including stroke [58,59]. IL-1β is rapidly released from the brain parenchyma after an ischemic event, triggering NADPH activation and thereby potentiating bradykinin-induced microvascular permeability [60]. Moreover, the release of bradykinin and IL-1β contribute to reactive oxygen species generation in the early stages of cerebral ischemia and reperfusion injury [19]. IL-1β application increases superoxide anion release from human endothelial cells and increases reactive oxygen species generation from mitochondria and NADPH oxidase in cultured retinal epithelial cells [61]. Additionally, bradykinin may act as a potential mediator of vascular leakage and pulmonary edema in patients with COVID-19 [12,13,14]. In this context, IL-1β release has been proposed as one of the key inflammatory mediators in COVID-19 [23,24,25] and could potentially exacerbate bradykinin-induced vascular permeability in these patients. Thus, employing drugs already in clinical use, such as simvastatin, could offer a therapeutic strategy for decreasing bradykinin- and/or IL-1β-induced pulmonary edema in patients with COVID-19.

In accordance with previous studies [19,62], we observed that concomitant application of IL-1β with apocynin, a specific inhibitor of NADPH oxidase, abolished the potentiation of bradykinin-induced microvascular permeability by IL-1β (Figure 5). Apocynin rapidly prevents the assembly of NADPH oxidase, by blocking the cytosolic subunit p47phox translocation to the cell membrane [37]. Furthermore, apocynin had no effect on simvastatin pretreated rats, suggesting that simvastatin pretreatment was sufficient to prevent the assembly of NADPH oxidase induced by IL-1β (Figure 6C). Pretreatment with simvastatin was effective in inhibiting IL-1β actions on bradykinin-induced permeability, suggesting that protection afforded by simvastatin against microvascular hyperpermeability may in part be due to inhibition of Nox2. Furthermore, it has been reported that IL-1β alone rapidly (within 10 to 15 min of its application) increases superoxide release in both cultured endothelial cells [63] and retinal epithelial cells, with the latter study suggesting that NADPH oxidase activation was involved [61]. Similarly, we have also demonstrated that IL-1β itself results in a small permeability increase (see Figure 6A), which was abrogated by simvastatin. These findings strengthen the proposition that simvastatin pretreatment prevents IL-1β stimulation of ROS generation via Nox2 assembly. Nevertheless, additional studies are necessary to investigate whether other pro-inflammatory cytokines, such as IL-2 and IL-6, could also increase bradykinin-induced microvascular permeability and whether statins could modulate the profile of these cytokines. 

By inhibiting reactive oxygen species generation and reducing the NAD+/NADH ratio, statins will reduce cellular oxidative stress [64,65,66]. Thus, protective cardiovascular effects of statins may be directly associated with their cellular antioxidant properties, independent of the cholesterol-lowering effects of these agents. As statins have been reported to activate the redox sensitive transcription factor Nrf2 and upregulate the cytoprotective antioxidant enzyme HO-1 [29,30,31,32,33], we postulated that loss of IL-1β potentiation of bradykinin-induced permeability may be a consequence of enhanced HO-1 activity. Notably, inhibition of HO-1 with SnPP did not restore the IL-1β-induced potentiation (see Figure 6B), suggesting that simvastatin probably acts via reducing NADPH oxidase activity. Statins have been reported to reduce NADPH oxidase activity by inhibiting isoprenylation of the protein Rac1 [28,66,67,68]. 

Isoprenylated Rac1 is essential for assembly of the NADPH oxidase enzymatic complex on the cell membrane [69]. In patients with heart failure, statin treatment reduces Rac1 function, NADPH oxidase activity and levels of reactive oxygen species [70], a finding consistent with our observation that simvastatin pretreatment reduces IL-1β/bradykinin mediated microvascular hyperpermeability. Reactive oxygen species have been reported to negatively regulate cell–cell adhesion controlled by intercellular adhesion molecules, such as VE-cadherin and β-catenin, which are linked to transmembrane molecules and the actin cytoskeleton. In addition to a role for reactive oxygen species, RhoA activation is important for bradykinin-induced permeability [71]. RhoA-GTP activation leads to actin cytoskeleton contraction, resulting in the breakdown of the endothelial barrier [72]. In this context, statins protect the endothelial barrier, reduce oxidative stress and inhibit isoprenylation and activation of RhoA and Rac1 [52,66]. 

In the present study, protection afforded by simvastatin against increased microvascular permeability in cremaster muscle venules in response to IL-1β and bradykinin may be associated with inhibitory effects on the assembly of NADPH oxidase subunit, leading to diminished NADPH oxidase mediated superoxide release. Although not investigated in the present study, other cytokines such as such as IL-6, TNF-α and IL17 may similarly potentiate bradykinin-induced microvascular permeability. It has been reported that simvastatin inhibits IL-6, IL-8 and IL-1β production in vitro [73,74], which may contribute to its protective role in cardiovascular diseases. We have now demonstrated that a key anti-inflammatory action of simvastatin is to prevent IL-1β mediated potentiation of bradykinin-induced permeability in skeletal muscle microvasculature. This study highlights a novel action by which simvastatin prevents the potentiation of bradykinin-induced permeability by IL-1β, possibly by targeting the assembly of NADPH oxidase subunits. The approach undertaken in this study was functional, and future studies focusing on the molecular pathways are needed to elucidate the exact mechanism by which simvastatin reduces NADPH oxidase assembly. 

## 5. Conclusions

Simvastatin could play an important role in the prevention and/or treatment of patients with a high predisposition to microvascular hyperpermeability mediated by pro-inflammatory cytokines potentiating the actions of bradykinin, with implications perhaps for vascular leakage and pulmonary edema.
